# Pili-Induced Clustering of *N. gonorrhoeae* Bacteria

**DOI:** 10.1371/journal.pone.0137661

**Published:** 2015-09-10

**Authors:** Johannes Taktikos, Yen Ting Lin, Holger Stark, Nicolas Biais, Vasily Zaburdaev

**Affiliations:** 1 Harvard University, School of Engineering and Applied Sciences, Cambridge, MA, United States of America; 2 Technische Universität Berlin, Institut für Theoretische Physik, Berlin, Germany; 3 Max-Planck-Institute for the Physics of Complex Systems, Dresden, Germany; 4 Brooklyn College of City University of New York, Department of Biology, Brooklyn, NY, United States of America; University of Lincoln, UNITED KINGDOM

## Abstract

Type IV pili (Tfp) are prokaryotic retractable appendages known to mediate surface attachment, motility, and subsequent clustering of cells. Tfp are the main means of motility for *Neisseria gonorrhoeae*, the causative agent of gonorrhea. Tfp are also involved in formation of the microcolonies, which play a crucial role in the progression of the disease. While motility of individual cells is relatively well understood, little is known about the dynamics of *N. gonorrhoeae* aggregation. We investigate how individual *N. gonorrhoeae* cells, initially uniformly dispersed on flat plastic or glass surfaces, agglomerate into spherical microcolonies within hours. We quantify the clustering process by measuring the area fraction covered by the cells, number of cell aggregates, and their average size as a function of time. We observe that the microcolonies are also able to move but their mobility rapidly vanishes as the size of the colony increases. After a certain critical size they become immobile. We propose a simple theoretical model which assumes a pili-pili interaction of cells as the main clustering mechanism. Numerical simulations of the model quantitatively reproduce the experimental data on clustering and thus suggest that the agglomeration process can be entirely explained by the Tfp-mediated interactions. In agreement with this hypothesis mutants lacking pili are not able to form colonies. Moreover, cells with deficient quorum sensing mechanism show similar aggregation as the wild-type bacteria. Therefore, our results demonstrate that pili provide an essential mechanism for colony formation, while additional chemical cues, for example quorum sensing, might be of secondary importance.

## Introduction

The micrometer-sized bacterium *N. gonorrhoeae*, also called gonococcus, is the Gram-negative pathogen, which causes the second most common sexually transmitted disease gonorrhea. These cells usually have a characteristic dumbbell shape of two merged spheres called a diplococcus. While lacking the ability to actively swim in a liquid, *N. gonorrhoeae* cells possess a form of motility that enables them to easily explore surfaces. This type of motility is called “twitching” [1, 2]. Twitching motility is powered by cycles of elongations and retractions of type IV pili, which are 5–9 nm in diameter thin filamentous appendages [3, 4]. The length of pili in general depends on the bacterial strain. Previously, the distribution of Tfp length for *N. gonorrhoeae* was reported to be well approximated by the exponential law with a mean of 0.9–1.2 *μ*m [5, 6], however, pili over 10 *μ*m length were also reported [7]. A pilus is assembled from within the cell from pilin subunits; there are ∼ 10–20 pili per cell [5, 6]. A pilus grows to a certain length and may attach to a surface or a pilus of another cell. As the disassembly of the pilus starts, the shortening of the attached pilus generates a pulling force, which propels the cell forward [8]. The force generated by Tfp is one of the strongest in the microbial world: the retraction of a single pilus can generate a force up to 100 pN, which corresponds to roughly 10,000 times the bodyweight of *N. gonorrhoeae* [9]. *N. gonorrhoeae* can also pull bundles of ∼ 10 pili cooperatively producing a total force in the nanonewton range [7]. Recently, the mechanism of twitching motility of individual *N. gonorrhoeae* cells was investigated in detail [5, 6, 10]. However, the mechanisms and dynamics of clustering of cells is still poorly understood.

A gonorrhea infection is the result of the presence and attachment of gonococci to the epithelial cells of the urethra. The bacteria do not only interact with the infected host cells by biochemical means, but also exert physical stress [11, 12]. As a consequence, the presence of pulling microcolonies triggers gene expression, rearrangements of the cytoskeleton, and ultimately the production of cortical plaques which are recruitment of various proteins underneath microcolonies creating a signalling hub [13, 14]. Higashi *et al*. investigated the clustering dynamics of gonococci in their natural environment on human epithelial cells and observed that within a few hours, symmetric, nearly spherical microcolonies formed, which were still motile [11]. Mutants deprived of the ATPase PilT still possess pili, but these pili can not be retracted. These mutants can still adhere to epithelial cells but their lack of retraction forces hinders the formation of the cortical plaques [15]. Instead of spherical microcolonies, *pilT* mutants form irregular aggregates incapable of rearranging host cells cytoskeleton [16]. Mutants that entirely lack pili or possess certain modifications of the primary sequence of the pilin main subunit show no aggregation [17]. A recent study also observed that the absence of oxygen can change the aggregative behaviour of *N. gonorrhoeae* [18]. These observations indicate that the clustering of cells is strongly dependent on the pili interactions and the ability of pili to retract.

To understand the mechanisms and dynamics of microcolony formation, we experimentally investigate the aggregation of wild-type *N. gonorrhoeae* cells on flat surfaces. While the mechanical role of pili in cell-to-cell interactions was established long time ago, it is not clear if there exist additional mechanisms at play that facilitate the clustering of cells and if pili dynamics alone can quantitatively explain the clustering behavior. In this work, we show that the pili-induced attraction between cells is *sufficient* for explaining the clustering dynamics of gonococci. Our results strengthen the hypothesis that even though other clustering mechanisms, such as for example quorum sensing or chemotaxis, cannot be ruled out completely, their effect is of secondary importance with respect to pili mediated interactions. We propose a simple theoretical description of the clustering process, where pili-pili cell interactions is the sole mechanism of aggregation. This model quantitatively reproduces the experimentally measured characteristics of clustering, such as area fraction, average size, and the number of cell clusters. To demonstrate the robustness of our approach, we use our model to describe the experimental data for clustering occurring on two different types of surfaces.

## Results

### Clustering dynamics

**Fig 1:**
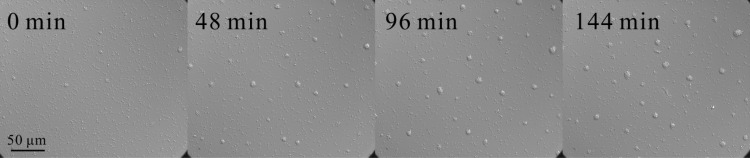
Clustering of *N. gonorrhoeae*. The snapshots of clustering on a rectangle of size 277 *μ*m × 234 *μ*m are taken at times *t* = 0, 48 min, 96 min, and 144 min (from left to right), and illustrate the aggregation process.

**Fig 2:**
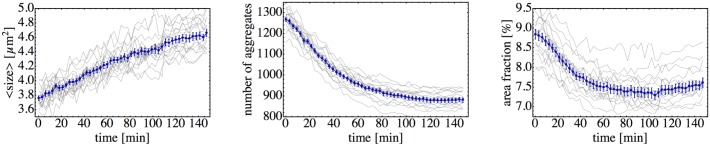
Quantification of *N. gonorrhoeae* colony formation on a glass surface. Mean cluster size, number of aggregates, and area fraction are plotted as functions of time; the curves and error bars (standard error of mean) are obtained from an ensemble average over 17 experimental realizations, which are all plotted as thin curves.

In S1 Video, we provide the movie of a representative clustering process; in Fig 1 several snapshots of the clustering are shown. After ∼ 2.5 hours of observation, that is, at the end of the experiment, we obtain a spatial distribution of circular, symmetric microcolonies. We focus on the time evolution of three observables: The mean cluster size, the number of aggregates, and the area fraction covered by the cells. By the cluster or aggregate we understand any compact and isolated body of cells we observe on a surface (see details of image analysis in Materials and Methods section). This formal definition includes single cells as clusters of the smallest possible size [19]. So it can be a single cell or a microcolony containing several hundreds of cells. We quantify the size of the cluster by the area it occupies on the surface. These observables are shown as functions of time in Fig 2. The mean cluster size gradually increases, whereas the total number of aggregates decays and finally reaches a steady state after *t* ∼ 100min. The area fraction is the total area occupied by all clusters, including individual cells, divided by the observation area (277 *μ*m × 234 *μ*m). It can be also calculated as the product of the mean cluster size and the number of aggregates, divided by the total area. The area fraction passes through a minimum at around ∼ 120min. It first decreases due to the formation of the three-dimensional microcolonies: a spherical colony resulting from the merger of two smaller spherical objects will have a smaller area projection than the sum of projected areas of the two original spheres. The area fraction then reaches a minimal value, as the merging process slows down in time, and after that it starts to continuously increase. At the same time, the total number of aggregates almost does not change, thus indicating that the increase of the area fraction has to be due to the cell growth.

**Fig 3:**
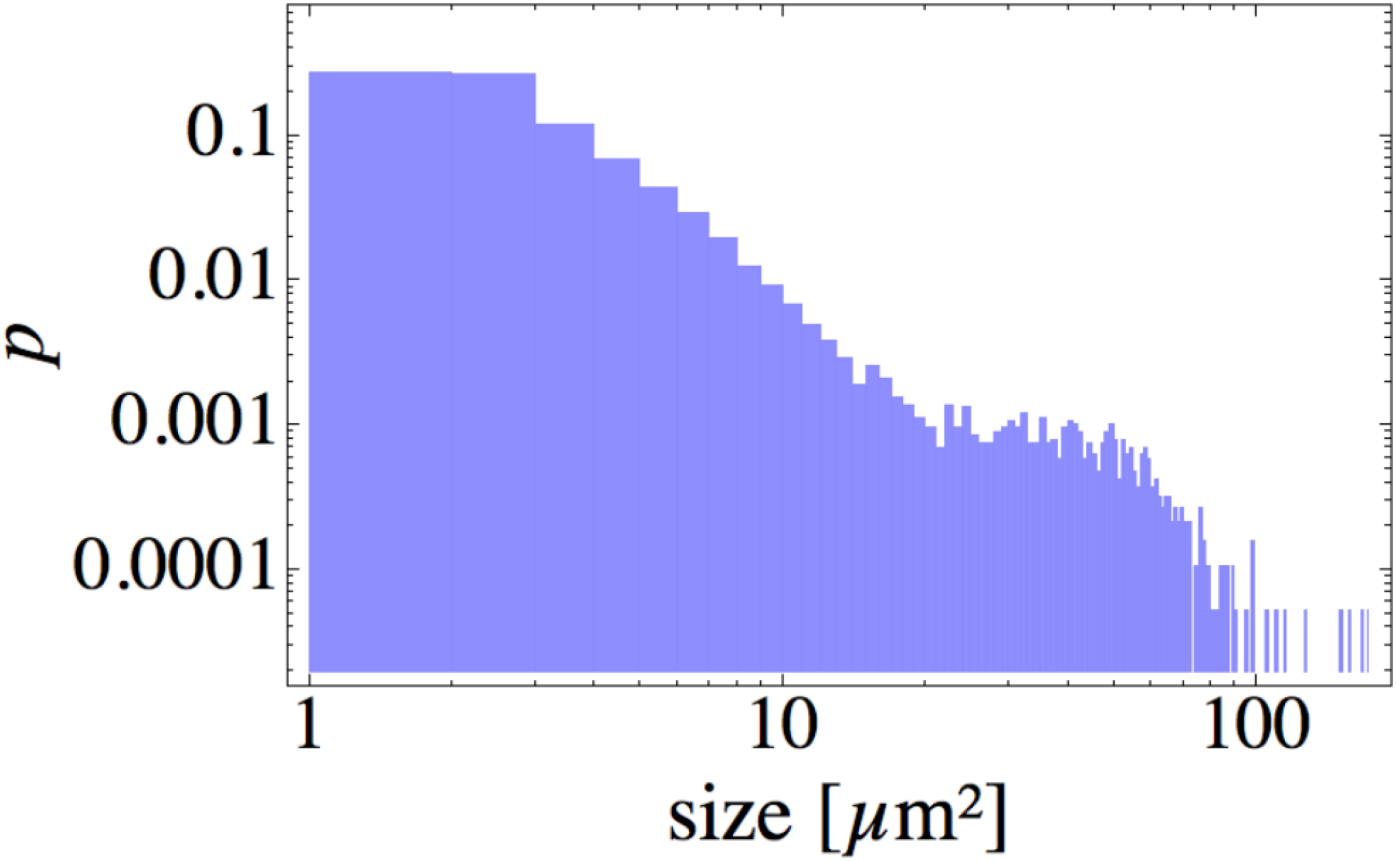
Probability density for the cluster size distribution. The distribution is obtained at the end of the clustering process (at the maximal time 147 min) from the same experiment, which provided the data for Fig 2. For a typical distribution of aggregates in space, we refer to Fig 1.

The normalized probability distribution of cluster sizes is shown as a double-logarithmic plot in Fig 3. It contains a large initial peak corresponding to relatively small aggregates of size 1–3 *μ*m^2^ (individual or a pair of cells). In addition, we observe the second peak at ∼ 30–50 *μ*m^2^; it corresponds to the average size of the microcolonies, which are also clearly visible in Fig 1. The large standard deviation 9.1 *μ*m^2^, as compared to the mean cluster size of 4.6 *μ*m^2^, reflects the width of the distribution.

To verify the central role of pili in aggregation process we looked at the △*pilE* mutant with a deleted main pilin subunit and thus deprived of pili in our experimental conditions. In agreement with the previous work [17] we saw no formation of microcolonies by bacteria lacking pili (see S1 Fig).

### Model

We construct a model, based on the diffusion and aggregation of clusters, to understand the dynamics of the experimentally observed clustering of *N. gonorrhoeae* cells. In this model, microcolonies are considered to be spheres that move on a flat surface and appear as disks on a two-dimensional projection. Each particle or a bacterial aggregate *i* is represented by its position vector **r**_*i*_ = (*x*_*i*_, *y*_*i*_) and radius *a*_*i*_. We assume that the motion of each particle is described by an overdamped Langevin equation (well known as a description of Brownian motion of a tracer particle in a fluid),

$$\begin{array}{c} \frac{\text{d}}{\text{d}t}\;r_{i}\left(t\right)=\sqrt{2D_{i}}\;\Gamma_{i}\left(t\right)\;. \end{array}$$ (1)

In the overdamped regime, friction force is proportional to velocity (left hand side of Eq (1)) and random forces (right hand side of Eq (1)) are dominating over the inertia of a particle. That is why there are only these two terms in the equation above. In the case of the Brownian particle, the random forces are of thermal origin and friction is the hydrodynamic friction of an object moving in a fluid. In our case, the Langevin equation has a phenomenological nature. Random forces are the result of active pili retraction events (and not thermal forces), that lead, on a long time scale, to the diffusive motion of cells and clusters. Mathematically, **Γ**_*i*_(*t*) is modeled as an uncorrelated Gaussian white noise (stochastic process). It is a vector with zero mean 〈**Γ**_*i*_(*t*)〉 = **0** and its components (denoted by indexes *α* and *β*) are independent and uncorrelated for any two moments of time *t* and *t*′ if *t* ≠ *t*′: ⟨Γ_*α*_(*t*) ⋅ Γ_*β*_(*t*′)⟩ = *δ*_*αβ*_ *δ*(*t*−*t*′), as expressed via the delta-function. The pre-factor $\sqrt{2D_{i}}$ characterizes the strength of the random force and determines the long-time diffusion constant.

In general, the diffusion coefficient *D*_*i*_ of each aggregate depends on its radius *a*_*i*_, as we see in experiments.

### Diffusion coefficient as a function of cluster size

**Fig 4:**
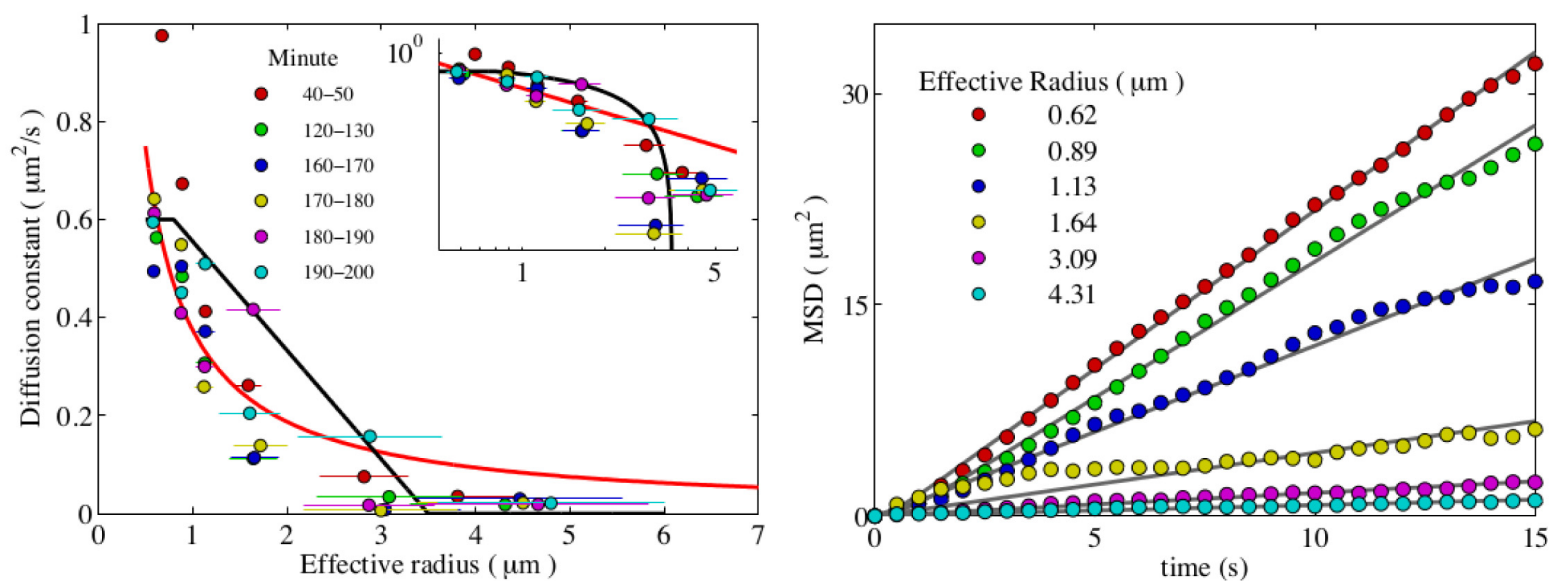
Cell motility as a function of the cluster size. Left: Diffusion coefficient *D* as a function of the cluster radius *a* for the glass surface. The thick black line is the linear function from Eq (2) with *D*_0_ = 0.6 *μ*m^2^ s^−1^, *a*_*s*_ = 0.8 *μ*m, and *a*_cut_ = 3.5 *μ*m. For comparison, the red line is the Stokes-Einstein scaling ∼ *a* ^−1^. Inset: Same data in a double-logarithmic plot. Horizontal error bars are the standard deviation of the cluster sizes in the corresponding bin. Right: The MSDs of binned cluster sizes are plotted versus time *t*; after fitting these curves with linear functions, their slopes yield the corresponding diffusion coefficients.

For a freely diffusing, spherical Brownian particle, the Stokes-Einstein relation determines the dependence of the diffusion constant *D* on a particle radius *a* as *D* = *k*_B_ *T*/(6*πηa*), where *η* is the viscosity of the fluid, *T* is temperature and *k*_B_ is Boltzmann’s constant. We decided to test if this approximation holds for our data. For that we tracked the clusters of different sizes and after fitting their mean squared displacement (MSD) as a function of time with a linear relation we obtained the estimates for their diffusion coefficients. To quantify the microcolony size we use the area *A* it covers in the image and introduce an effective radius calculated as $a=\sqrt{A/\pi }$. We show the results in Fig 4.

One distinct feature of these measurements is that larger clusters almost do not move, starting to show the signatures of subdiffusion, when the MSD scales slower than linear in time (there are not enough trajectories of larger colonies to make a more definite statement on the nature of diffusion in this regime, see the double logarithmic plot in S2 Fig). For comparison, we show the Stokes-Einstein relation (red line), which even for very large cluster sizes predicts a non-zero diffusion constant. From the point of view of agglomeration and implications for modeling there is a significant difference. In experiments, after growing above a certain size colonies lose their ability to move. Therefore after some transient dynamics when cells and smaller colonies move around and aggregate, a final state with multiple immobile colonies will be reached. The colonies will then grow only due to cell growth and division, but not via merging events. This is exactly what we see in our experimental data. At long times the area fraction starts to grow, while the number of clusters remains almost constant, and there are multiple large colonies in the field of view.

In case of Stokes-Einstein relation, even very large aggregates are still able to move. Eventually the final state of such agglomeration process would be a single huge cluster of particles. Therefore, for modeling it is important to capture the vanishing diffusion constant. We use the simplest approximation of the experimental data by a linear function that decays and terminates at a certain critical cluster size *a*_cut_ (see black line in Fig 4).

$$\begin{array}{ccc} D(a) & = & \left\{ \begin{array}{cc} \;D_{0},\;\; & \;\;a\leq a_{s},\\ \;D_{0}\;\frac{a_{\text{cut}}-a}{a_{\text{cut}}-a_{s}},\;\;a_{s}\leq & \;\;a\leq a_{\text{cut}},\\ \;0, & \;\;a>a_{\text{cut}}; \end{array}\right. \end{array}$$ (2)

Single cells with a size below *a*_*s*_ = 0.8 *μ*m have the largest diffusion constant *D*_0_ = 0.6 *μ*m^2^ s^−1^, consistent with recent measurements [10]. *D*(*a*) then linearly decays to zero and vanishes for *a* > *a*_cut_, with the cut-off radius *a*_cut_ = 3.5 *μ*m,

The exact mechanism of vanishing diffusion for bacterial microcolonies still needs to be investigated, but we can offer a qualitative explanation of this phenomenon. Recently, when analyzing at the motility of single *N. gonorrhoeae* cells, it was shown that bacteria employed multiple pili to propel themselves along the surfaces [5, 6]. In the model, multiple pili could pull cooperatively, but also against each other. When pili are competing, the cell is trapped and does not move. It was shown that by increasing the amount of pili in the model, the trapping time became longer. We note that large clusters of cells still rely on pili to move. For larger clusters the number of pili increases roughly proportionally to the surface area adjacent to the substrate. We can speculate that due to a large number of competing pili, bigger microcolonies are trapped most of the time. To quantify and check this hypothesis is a subject of further research. It would also offer a guide for a better fit of the dependence of the diffusion constant on the cluster size, but for now we just use the simplest linear approximation.

### Cell growth

To take into account the growth of cells, we introduce the growth rate *λ* with which the total bacterial mass *M* increases exponentially according to

$$\begin{array}{c} M\left(t\right)=M_{0}\;e^{\lambda t}. \end{array}$$ (3)

Using the mass density *ρ*_*m*_ and writing *M* ∝ *ρ*_*m*_ *a* ^3^, the radius *a*(*t*) grows like

$$\begin{array}{c} a\left(t\right)=a_{0}\;e^{\frac{\lambda }{3}t}, \end{array}$$ (4)

and the area fraction ∝ *a* ^2^ increases exponentially with the rate $\frac{2}{3}\lambda$. In the following, each sphere of our model with radius *a*(*t*) will grow according to $\frac{\text{d}}{\text{d}t}\;a(t)=\frac{\lambda }{3}\;a(t)$, which is equivalent to Eq (4). Hence, we need to estimate the growth rate *λ*.

According to Fig 2, the number of aggregates is almost constant at times larger than 120min. Therefore, the increase of area fraction has to be due to the cell growth. Within an interval of Δ*t* = 27min, the area fraction changes from about 7.44% to 7.62%, which corresponds to an increase of 2.42%. Solving $e^{\frac{2}{3}\;\lambda \Delta t}∼1.0242$ for *λ*, we can estimate the growth rate

$$\begin{array}{c} \lambda ∼2.2\times 10^{-5}\;{\text{s}}^{-1}\;\text{or}\;\lambda^{-1}∼752\min. \end{array}$$ (5)

This value is consistent with an optical densitometry (OD) measurement; it provided the estimate that the cell density increases by about 20–30% within *T* ∼ 3 hours. Solving Eq (3) for *λ*, *e* ^*λT*^ = 1.2–1.3, gives *λ* ^−1^ ∼ 686–987min. We should note that such a low observed growth rate is due to the fact that the bacteria are grown in DMEM media without CO_2_. These are the conditions and media, which allow for investigation of the interaction of bacteria and human cell culture, but they are not optimal for exponential bacterial growth [20]. Low growth rate is also beneficial for our study, as we want to focus on the role of pili-mediated motility and interactions. As a result we can temporarily decouple the effects of active merging and divisions of cells. Although we obtained the value of the growth rate from the optical density measurements, we treat the growth rate as a fit parameter. The reason for that is that the exact conditions of the cells growing in colonies on a surface would never be identical to those of the OD measurements. For example, in OD measurements, cells need to be resuspended, thereby giving them access to fresh nutrients, whereas cells inside of a larger colony may feel the nutrient limitations. One example of such behavior is the difference in growth of a biofilm and planktonic cells [21]. Therefore, a better estimate of the growth rate can be obtained when matching the complete theoretical model and the experimental data on clustering.

### Merging rule for pili-mediated interaction

In the pioneering study of Merz, So, and Sheetz [3] that identified pili as a driving force of twitching motility, an interesting experiment was performed. By using an optical tweezers setup, a cell was brought in the vicinity of a cluster of cells. It then was observed that the cell was actively pulled toward the cluster. That experiment demonstrated that pili were also responsible for cell-cell interactions. When two clusters of cells are close to each other, so that their pili can interact, a similar process is happening. The pili establish a link and actively pull colonies together which then merge into a larger aggregate.

**Fig 5:**
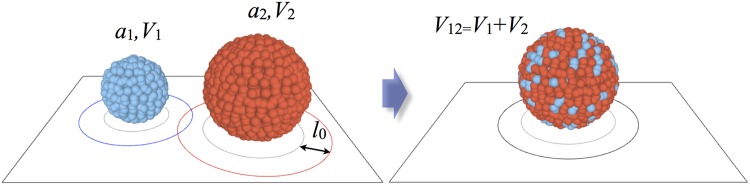
Model for the merging of bacterial clusters. Each spherical cluster consists of a certain number of gonococci and is characterized by its radius *a* and volume $V=\frac{4}{3}\pi a^{3}$. The two sketched aggregates merge after an exponentially distributed waiting time, if the corresponding pili shells of the width *l*_0_ overlap. After aggregation, the total volume of cells is conserved (see also the explanation in the text). Note that the extent of the actual mixing of cells from two different clusters is not known and here should be only considered as an illustration.

We propose a merging rule that respects mass and volume conservation in three dimensions. Note that mass conservation is the reason why each cell aggregate on the surface is better represented as a three-dimensional sphere instead of a two-dimensional disk. To mimic a uniform distribution of pili on the cell surface, we introduce a “shell” of width *l*_0_ around each particle, where *l*_0_ is the average pili length. The pili-mediated merging of colonies is sketched in Fig 5 and occurs as follows. If the shells of two clusters, located at positions **r**_1_ and **r**_2_ with radii *a*_1_ and *a*_2_ respectively, are overlapping,

$$\begin{array}{c} |r_{1}-r_{2}|<a_{1}+a_{2}+2l_{0}\;, \end{array}$$ (6)

both clusters can merge into a new sphere with the radius

$$\begin{array}{c} a_{12}=\sqrt[{3}]{a_{1}^{3}+a_{2}^{3}}\;, \end{array}$$ (7)

whose center-of-mass **r**_12_ is given by the weighted average

$$\begin{array}{c} r_{12}=\frac{a_{1}}{a_{1}+a_{2}}\;r_{1}+\frac{a_{2}}{a_{1}+a_{2}}\;r_{2}\;. \end{array}$$ (8)

We should note that the volume conservation in three dimensions implies that, in a system without cell growth, the area fraction during clustering is not conserved. This leads to initial decay in the total area fraction occupied by cells. As the next step, we take into account that clustering does not happen instantaneously, as the growth of pili requires time and the pili-pili interactions have a stochastic nature. In other words, the cells need some “time for shaking hands” and merging becomes a probabilistic process. For two aggregates in contact, we therefore introduce a waiting time as a random variable with exponential distribution and mean waiting time *T*_*w*_. If two aggregates satisfy Eq (6), the probability *P* of merging within a small time interval Δ*t* ≪ *T*_*w*_ is time-independent and becomes

$$\begin{array}{c} P=\Delta t/T_{w}. \end{array}$$ (9)

**Fig 6:**
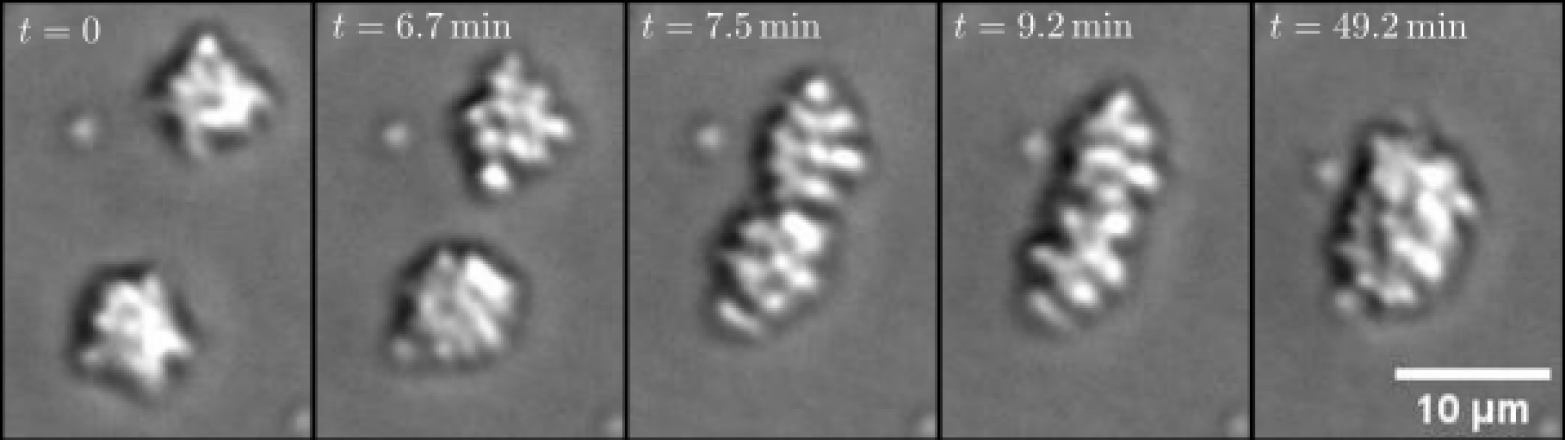
Merging of two clusters. Two large microcolonies merge into an aggregate that finally tends to an almost spherical shape.

We will determine *T*_*w*_ to match simulation and experimental results, which is of the order of minutes. Altogether, exponentially distributed waiting times imply that merging occurs only with a certain probability, and two nearby aggregates can also separate again by diffusion without having merged. The first picture of Fig 6 shows two large microcolonies whose distance between the outer edges is ∼ 8 *μ*m. We notice that both aggregates remain relatively immobile for quite a long time until the cells attract and start to merge into one larger cluster. It might be explained by the fact that a certain time is required to grow pili that are long enough to reach from one cluster to another but also to have a sufficiently high number of them to be able to pull the clusters together. Therefore the process of merging is not a trivial process, and depends on various parameters: distance between the clusters, their sizes, and the time they spend close to each other. In our model we condense these dependences in a single time constant *T*_*w*_.

Fig 6 also shows a tiny aggregate, the small spot left from the upper microcolony, which hardly moves, and in particular, does not merge with the nearby cluster. The large number of non-merging, small aggregates is also visible in Fig 1 and strongly dominates the size distribution in Fig 3. The exact nature of those immobile cells is not entirely clear. It could be dead cells, or cells carrying a mutation in one of the genes responsible for the pili operation. In principle, those cells could be removed by image-analysis tools; however, for several reasons that could not be done reliably without affecting properly moving cells. First of all, not all individual cells remain immobile for the whole duration of the experiment; after some long but transient time they might start to move again (and vice versa). As shown in [6], such trapping might be even a natural component of cell motility. Secondly, some of the immobile cells get incorporated (stick to) into larger colonies when ran over by them. Therefore we do not separate moving and immobile cells on the level of data, but instead introduce a certain fraction of immobile particles *m*_*p*_ in our simulations and treat this fraction as an additional parameter. These particles, however, are allowed to grow as the normal cells. These “passive” cells, depending on the experimental realization, may amount to ∼ 60% (in the number) of all cells.

## Comparison of the model and data

We perform numerical simulations of our model with periodic boundary conditions on a rectangular surface of the same size as in the experiment (277 *μ*m × 234 *μ*m), and for the same duration of ∼ 150min. The initial configuration for *t* = 0 is generated from a representative sample of the experimental data (see the first frame of Fig 1), and we approximate each aggregate of area *A* by a sphere of radius $a=\sqrt{A/\pi }$. Simulations start with a total number of *m*_0_ = 1291 aggregates, and we randomly select *m*_*p*_ = 796 particles as the “passive” ones. This number is, in fact, also a parameter in our model. As its first approximation we use the number of clusters at the end of the experiments and then gradually vary it (decrease) in order to get the best match of the simulations and data. The diffusion coefficient of cell aggregates is modeled using the linear estimate, as presented in Fig 4.

**Fig 7:**
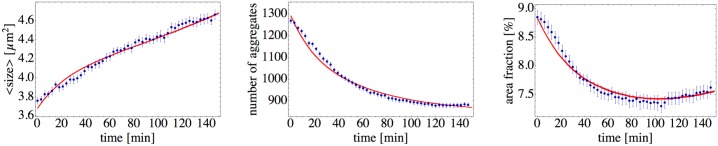
Comparison of the model and data. Red curves show the simulation results, which are compared to to the experimental data points. Error bars are showing the standard error of mean. The initial conditions for simulations are taken from an experiment with 1291 particles, with 495 “active” and *m*_*p*_ = 796 “passive” particles. Diffusion constant as a function of cluster size is given in Fig 4 *D*_0_ = 0.6 *μ*m^2^ s^−1^, with minimal and cut off cluster sizes set to *a*_*s*_ = 0.8*μm*, and *a*_cut_ = 3.5*μm*. The pili length *l*_0_ = 1.6 *μ*m, growth rate *λ* = 4.2 × 10^−5^ s^−1^, and a delay time for the establishment of pili-pili contact *T*_*w*_ = 18.3min.

To match the experimental data, we adjust the waiting time as *T*_*w*_ = 18.3min, growth rate *λ* = 4.2 × 10^−5^ s^−1^ and the pilus length as *l*_0_ = 1.6 *μ*m. Note that the pili length has to have a value around 1–2 microns to comply with previous data, and the growth rate has to be close to the independent OD measurements. Therefore the only parameter, for which we have no prior knowledge, is the waiting time *T*_*w*_, which characterizes the cell-cell interactions. The simulation results are shown in Fig 7: The curves for the mean cluster size, the number of aggregates, and the area fraction demonstrate a nice agreement between experiment and simulation. In particular, our model reproduces the decrease in the number of aggregates approaching a constant value for large times. Together with the increasing mean cluster size it results in the shallow minimum of the area fraction at intermediate times. The initial decrease in the area fraction is the clear indication of the three-dimensional nature of clusters and that their merging is governed by volume rather than by area conservation.

### Clustering on a plastic surface

**Fig 8:**
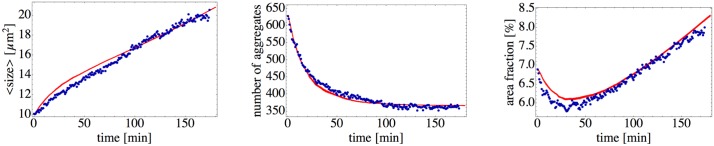
Quantification of clustering on a plastic surface. Comparison of experimental data (blue) to simulation results (red curves). The initial conditions for simulations are taken from the experiment with 629 particles, with 349 “active” and *m*_*p*_ = 280 “passive” particles. The motility-related parameters are *D*_0_ = 0.3 *μ*m^2^ s^−1^, *a*_*s*_ = 0.9 *μ*m, and *a*_cut_ = 3.2 *μ*m (see S3 Fig), and the growth rate is determined as *λ* = 6.7 × 10^−5^ s^−1^. Using the same pilus length *l*_0_ = 1.6 *μ*m as before, we determine the mean waiting time to be *T*_*w*_ = 4.6 min.

To test the robustness of the model, we perform similar experiment as before but on a plastic surface (see S3 Fig). As seen in S4 Fig, there is less data than for the glass surface. However, we still can capture the main feature of vanishing diffusion and approximate the size-dependent diffusion coefficient by a linear function from Eq (2), with slightly modified parameters. We estimate a bacterial growth rate to be *λ* = 6.7 × 10^−5^ s^−1^, adjust the proportion between active and passive particles, and by fitting the experimental curves find the waiting time parameter to be *T*_*w*_ = 4.6min. As a result, simulations of the model reproduce the experimental data set for plastic surface as well, see Fig 8. Although the model works with the same pilus length *l*_0_ = 1.6 *μ*m for clustering on both the glass and the plastic surface, the characteristic interaction time should be changed. It has a simple explanation, as we do not expect the changes in the surface properties to effect the pili length. In contrary, as the characteristic time required for two clusters to aggregate is determined by the balance of the cell-surface, and cell-cell (or pili-pili) interactions, we expect *T*_*w*_ to change for a different kind of surface. To demonstrate how sensitive is the model is to the variation of the main parameters we provide a series of plots in S5–S7 Figs where the interaction rate *T*_*w*_, the ratio of active to passive particles, and the pili length *l*_0_ are changed independently.

The observed agreement between theory and experiment for two distinct surface types further justifies the assumption that clustering in *N. gonorrhoeae* is mainly driven by pili interactions.

## Discussion

The prerequisites for clustering of cells are the surface attachment and motility, which, in case of *N. gonorrhoeae*, are both provided by Tfp. However, what is the exact mechanism that allows the cells to find each other? One of the well known scenarios of agglomeration in the bacterial world is driven by auto-chemotaxis, when individual cells secrete a signalling chemical and bias their motility to the gradient of its concentration. Such systems are known to produce dense, symmetrical clusters [22, 23]. Motivated by the existing knowledge on Tfp and their role in the single cell motility, we hypothesised that these long filaments may significantly enhance the interaction radius of motile cells and serve as the predominant mechanism for aggregation. To test this hypothesis, we studied the process of cluster formation by *N. gonorrhoeae* bacteria on two different kinds of surfaces. The analysis of our experimental data focuses on the time evolution of the mean cluster size, total number of clusters, and area fraction covered by the cells. We suggest a simple theoretical model, which assumes the pili-pili interaction as the exclusive mechanism for cell aggregation and takes most of other parameters directly from the experimental data. The model also features the diffusive motion of clusters, which depends on their size, cell growth, and a merging rule that accounts for mass (volume) conservation. Numerical simulations of the model agree well with the experimental data. These results strongly suggest that indeed the pili-induced interaction between the cells could be a predominant mechanism of the cluster formation. There are another two important mechanisms that can facilitate the clustering of cells. One of them is chemotaxis. Our search in the MIST2.2 data base [24] shows the absence of known chemotactic systems in the gonococcal genomes. Evaluation of the genome of the strain used in this present study through an automated annotation tool as RAST [25] also leads to the absence of known chemotactic systems. Another system implicated in the collective interactions of cells is the quorum sensing system [26]. We studied the formation of colonies in the quorum sensing mutant of *N. gonorrhoeae* (△*luxS* mutant) and found the clustering to be identical to the wild type bacteria (see S8 Fig for a snapshot of the final stage of aggregation and the corresponding cluster size distributions). We could have tested more mutants, but we would never be sure that there is no another alternative cue that we would be missing. Therefore we believe that in the context of possible but unknown alternative mechanisms, the approach we took with theoretical modeling is the practical way to address the problem of clustering in *N. gonorrhoeae* cells in a quantitative way. The model has a very basic background of stochastic processes, which can be modified or generalized to make it applicable to similar bacterial or other individual based clustering problems. It will be interesting to combine a more detailed microscopic description of twitching motility on a single cell level [6, 10, 27] and collective behavior [28–30].

Though the current theoretical model already explains the experimental data with respect to main clustering characteristics, the present study offers interesting experimental and theoretical challenges. It would be interesting to accurately relate the motion of clusters to their size and see if the diffusive motion of large clusters is indeed normal or anomalously slow. Further progress can be made on the model assumptions, as for example to specify the dependence of the characteristic waiting time for merging on the distance and cluster sizes. Another challenging problem is to understand the observed clustering dynamics in terms of kinetic models in the spirit of statistical physics [31]. Such a mesoscopic description might, for instance, be derived from the presented model and yield the cluster size distribution as a function of time which then can be related to the experimental measurements.

We believe that our results highlight the importance of Tfp in the clustering dynamics of *N. gonorrhoeae* bacteria and therefore potentially lead to a better understanding of the initial stages of gonorrhea infection. It should be noted that clustering of bacteria on surfaces in a broader context may be viewed as the initial stage of biofilm formation [32–34]. The formation of *N. gonorrhoeae* microcolonies can be seen as the first steps towards the formation of biofilms in general. Elucidating the biophysical parameters and the use of retraction forces in this process might be opening much needed new ways to prevent the formation of microcolonies and potentially control *N. gonorrhoeae* proliferation.

## Materials and Methods

### Cell culture and imaging

Our experiments with *N. gonorrhoeae* are performed with the MS11 strain and the same pilin sequence that was crystallized in Craig et al. [35]. The deletion mutant of the main pilin subunit PilE is a gift from the So lab [26]. The deletion mutant of the quorum sensing protein LuxS was obtained by allelic replacement of the *luxS* opening reading frame by a chloramphenicol cassette. Bacteria are grown on GCB (gonococcal broth) medium agar plates in an incubator at 37°C and 5% CO_2_ for 16 to 20 hours, 5 *μ*g/ml of erythromycin was added in the case of the *pilE* mutant and 5 *μ*g/ml of chloramphenicol was added in the case of the *luxS* mutant. Before the experiment, cells are resuspended in liquid GCB medium and their density is assessed by an optical densitometry measurement. Cells are then diluted in DMEM liquid medium at the concentration of 5 × 10^7^ cells in 2 ml. For experiments on a glass surface this volume of 2 ml is added to a 35 mm diameter glass bottom dish. For the plastic surface the same volume is added to one well of a 6-well polystyrene plate with the same diameter of 35 mm. The number of 5 × 10^7^ bacteria on a circular area with 35 mm diameter yields an initial cell density of 0.052 *μ*m^−2^. By idealizing a bacterium as a disk of radius *a*_0_ = 0.7 *μ*m (and area $\pi a_{0}^{2}=1.54\;\mu {\text{m}}^{2}$) we estimate the initial area fraction of *ρ*_0_ ∼ 8%.

Bacteria are observed with an optical Nikon eclipse Ti microscope using DIC (60 × magnification) on a glass surface and an Olympus IX81microscope (40X magnification) on a plastic surface. Images are taken at a frame rate of 1/3–10 Hz, and the observation area typically comprises a region of 277 *μ*m × 234 *μ*m and 350 *μ*m × 262*μ*m for glass and plastic respectively; for demonstration, grayscale images as provided by the microscope are shown in Fig 1 and S2 Fig. To obtain a quasi-two-dimensional system, the bacteria are sedimented by centrifuging the plate at 2465 × *g* for 5min. The preparation of suspensions comprized of individual cells results in shearing off the pili from the cell body, so that a certain lag time of pili regrowth might be required before the cells start to move and interact normally. At the same time the microscope provides stable images only after approximately ∼ 30–40min of observation that are needed to fully thermally equilibrate the sample on the microscope. In our analysis we set the time *t* = 0 after the equilibration of the setup is finished (when no drift is measured). To provide a sufficiently reliable statistic analysis of the clustering dynamics on a glass surface, we captured 17 independent realizations of the same cell culture by imaging the corresponding number of distinct areas on the sample.

### Image analysis

The edges of the cells are first detected by the Canny edge detector, followed by dilation and erosion operations to “fill” the holes surrounded by detected edges. Thresholds and parameters of the operations are optimized to faithfully represent the morphology of the cells. When the contrast is low and the edges are not clear, instead of edge-detection, standard image processing by thresholding the intensity of the image are adopted. Since the intensity-thresholding method only partially detects the central part of the cells, nearby structures are labeled as a single cluster and a suitable dilation operation is imposed. For the estimate of the diffusion coefficients, identified structures in consecutive (2 Hz) frames were fed into a standard tracking algorithm, which minimizes the distance functional [36]. After the trajectories are binned by their corresponding time-averaged area of the corresponding cluster, the standard time-averaged mean-squared displacement, as a function of both cluster area and time, is calculated.

## Supporting Information

S1 Fig A mutant lacking pili does not aggregate. Two snapshots of the agglomeration process by a wild-type (right panel) and *pilE* mutant lacking pili (left panel) at the same conditions after 3 hours of observation. The mutant lacking pili is unable to form micro-colonies.
(EPS)

S2 Fig Mean squared displacement of clusters with different sizes. The same as Fig 4 but in a double-logarithmic plot.
(EPS)

S3 Fig Clustering on a plastic surface. The snapshots on a rectangle of size 350 *μ*m × 262 *μ*m are taken at times *t* = 0, 74.2 min, 174.5 min (from left to right). The clustering is analyzed in terms of the mean cluster size, number of aggregates, and area fraction, which is shown as the blue data points in Fig 8.
(EPS)

S4 Fig Motility on a plastic surface: Diffusion coefficient *D* as a function of cluster radius *a*. The thick dashed line is the linear function from Eq (2) with *D*_0_ = 0.31 *μ*m^2^ s^−1^, *a*_*s*_ = 0.9 *μ*m, and *a*_cut_ = 3.2 *μ*m. For comparison, the thin dashed line is the Stokes-Einstein scaling ∼ *a* ^−1^. The inset shows the corresponding mean-squared displacements versus time *t*, which are fitted by 4*Dt* (dashed lines).
(EPS)

S5 Fig Response of the model to the variation of the waiting time. *T*_*w*_ = 550s (orange line), *T*_*w*_ = 1650s (magenta line), and *T*_*w*_ = 1100s (red line). All other parameters are the same as in Fig 7.
(EPS)

S6 Fig Response of the model to the variation of the ratio between active and passive particles. *m*_*p*_ = 720 (orange line), *m*_*p*_ = 880 (magenta line), and *m*_*p*_ = 796 (red line). All other parameters are the same as in Fig 7.
(EPS)

S7 Fig Response of the model to the variation of the pili length. *l*_0_ = 0.8 *μ*m (orange line), *l*_0_ = 2.4 *μ*m (magenta line), and *l*_0_ = 1.6 *μ*m (red line). All other parameters are the same as in Fig 7.
(EPS)

S8 Fig Quorum sensing mutant shows the same clustering as the wild-type. Cluster size distribution after 3 hours of observation for the *luxS* mutant (left panel) and wild-type (right panel). The insets show representative snapshots of the corresponding clustering processes. The distributions are obtained from 20 different positions on the same sample for identical conditions for the mutant and the wild-type control.
(EPS)

S1 Video Clustering of *N. gonorrhoeae* bacteria. This video shows the process of cluster formation of *N. gonorrhoeae* bacteria on a plastic surface. Scale bar is 20 *μ*m and the duration of the movie covers 147 minutes.
(MP4)
